# Heel Pad Reconstruction With Medial Plantar Flap

**DOI:** 10.7759/cureus.13987

**Published:** 2021-03-19

**Authors:** Adriana S Langat, Wan Azman Wan Sulaiman, Siti Fatimah Noor Mat Johar

**Affiliations:** 1 Plastic and Reconstructive Surgery, Universiti Sains Malaysia School of Medical Sciences, Kota Bharu, MYS

**Keywords:** heel pad, medial plantar flap, flap reconstruction, outcome, functional, weight bearing, calcaneous, heel pad loss, heel pad deformity

## Abstract

The heel of the foot is covered by highly specialized thick, glabrous skin containing fibroadipose tissue with numerous fibrous septae traversing the subcutaneous tissue, which acts as a shock-absorbent and prevents shearing of the skin. The loss of heel pad would cause interruption of the propelling function of the foot during walking. Therefore, heel pad reconstruction is an important procedure for wound closure in the acute phase and also functional reconstruction in delayed cases. We report a case of heel pad deformity in a patient who presented to us with left heel pain and inability to fully bear weight, which has caused her walking difficulty, following a road traffic accident. She sustained a degloving injury of the left foot and an open fracture of left calcaneum with ruptured Tendon Achilles in which the wound was initially addressed with failed reverse sural flap and the wound was allowed to heal by secondary intention. Delayed heel reconstruction was carried out with a propeller medial plantar flap and split skin graft. Postoperatively, the patient had improved functional and esthetic outcome.

## Introduction

The heel of the foot is covered by highly specialized thick, glabrous skin containing fibroadipose tissue with numerous fibrous septae traversing the subcutaneous tissue to connect the skin to the plantar aponeurosis. These septae divide the subcutaneous tissue into small loculi that acts as a shock-absorbent and prevents the skin from shearing [1]. Therefore, the heel is an important integrated part for walking. It is subjected to more repetitive trauma and loading stress than any other part of the body. The loss of heel pad would cause the interruption of the propelling function of the foot during walking. Hence, heel pad reconstruction is an important procedure for wound closure in the acute phase and also functional reconstruction in delayed cases.

Defects of the heel most commonly result from acute trauma, chronic wound, or tumor excision. Most cases of isolated soft tissue injuries result from a degloving type of injury. In the era of modern medicine, the demand for limb salvage surgery has been on a rise, in which the main goal is to achieve long-term functional stability with minimal morbidity in a short time [2]. We present a case of delayed heel pad reconstruction with its functional outcome.

## Case presentation

A 21-year-old female presented to us with complaints of left heel pain on walking and often develop ulceration over her left heel. She was unable to fully bear weight over the left heel, hence resulting in walking difficulty. She had a history of road traffic accident with a degloving injury of the left foot and an open fracture of left calcaneum with Tendon Achilles partial rupture. The wound was initially addressed with reverse sural flap of the left heel; however, it was complicated with infection and was removed. The Tendon Achilles was repaired, and the wound then healed with secondary intention.

**Figure 1:**
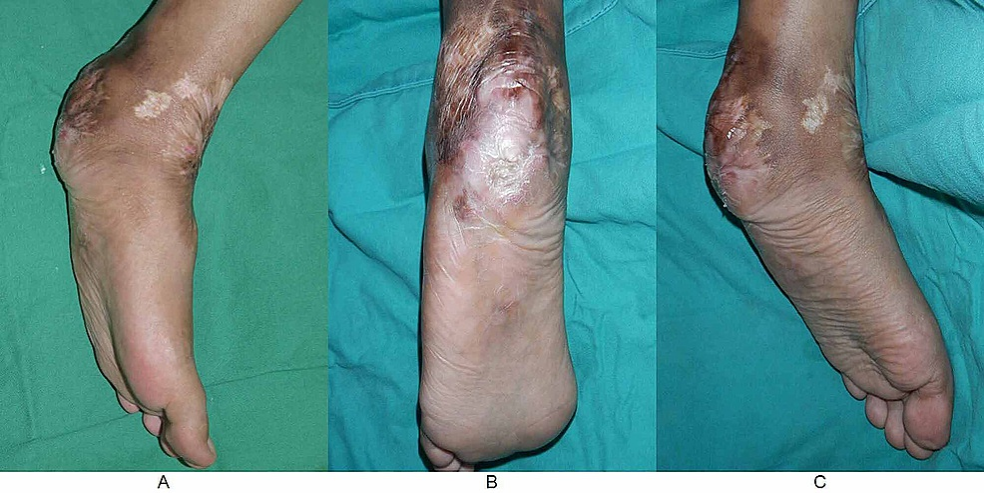
Preoperative photos. Note the loss of feel pad with obvious bony prominence. Note the healed scar from previous surgery

Examination revealed a healed scar over the heel; however, there was loss of heel pad with bony prominence felt at the heel (Figure 1). The range of movements of the ankle was full.

**Figure 2:**
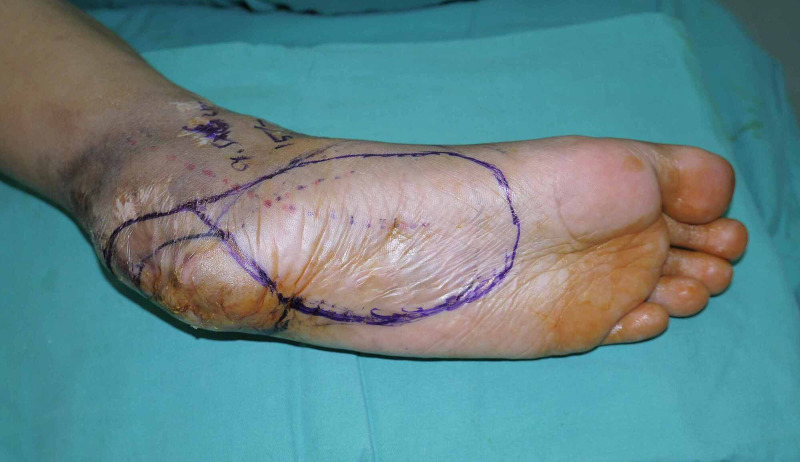
Preoperative planning of the flap

**Figure 3:**
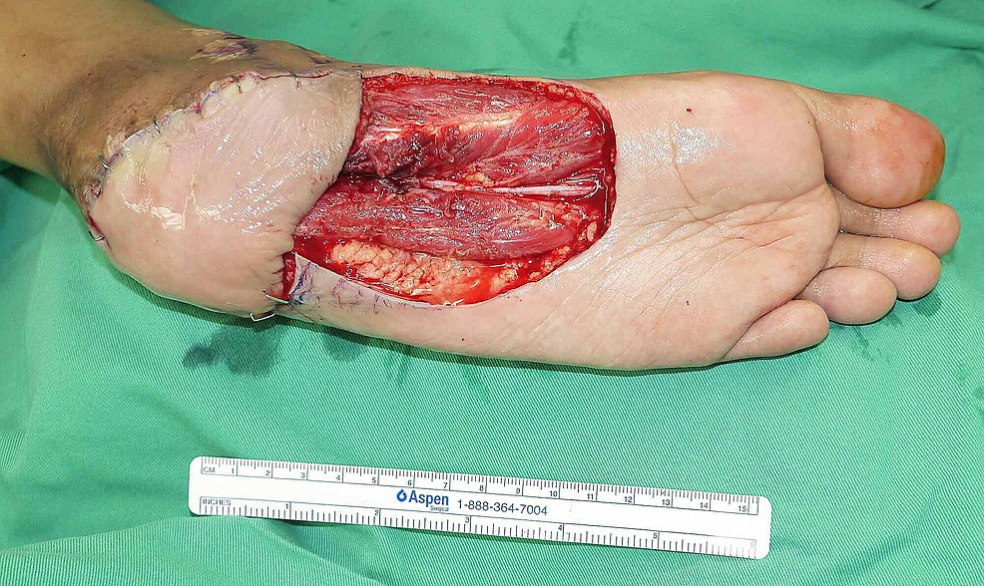
After flap harvest and inset, leaving behind donor-site defect exposing the flexor digitorium brevis and flexor digiti minimi, which was then covered with split skin graft

The patient underwent left heel pad reconstruction with propeller medial plantar flap. The heel scar was debrided, leaving behind a 6.5 x 6.5 cm defect. Medial plantar flap was designed according to the defect size from the ipsilateral foot (Figure 2). Flap was raised under tourniquet control and with loupe magnification. Dissection was done at the subfascial plane and transposed into the defect, based on the medial plantar artery (Figure 3). Perforator vessels were encountered best at the head of flexor hallucis brevis. The location of perforator was confirmed using a hand-held Doppler. The secondary defect was then covered with split-thickness skin graft, harvested from the ipsilateral thigh. A backslab was applied postoperatively and was later placed in a thermoplastic splint.

**Figure 4:**
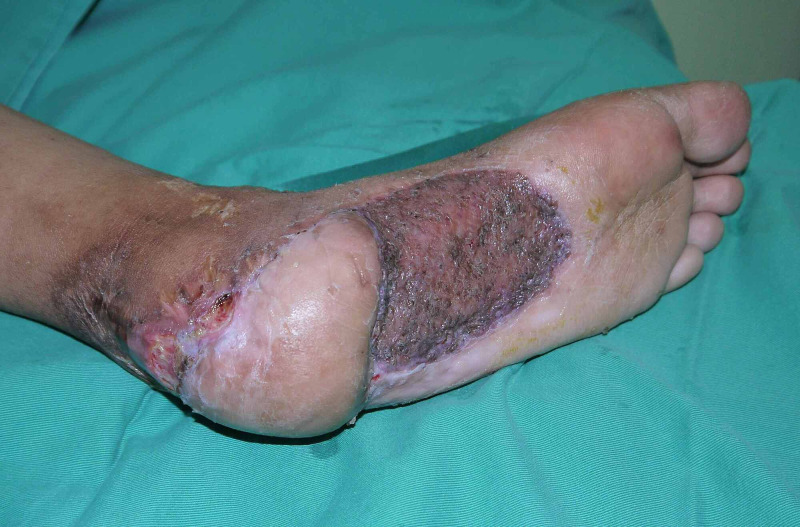
Healed donor site one month after surgery

**Figure 5:**
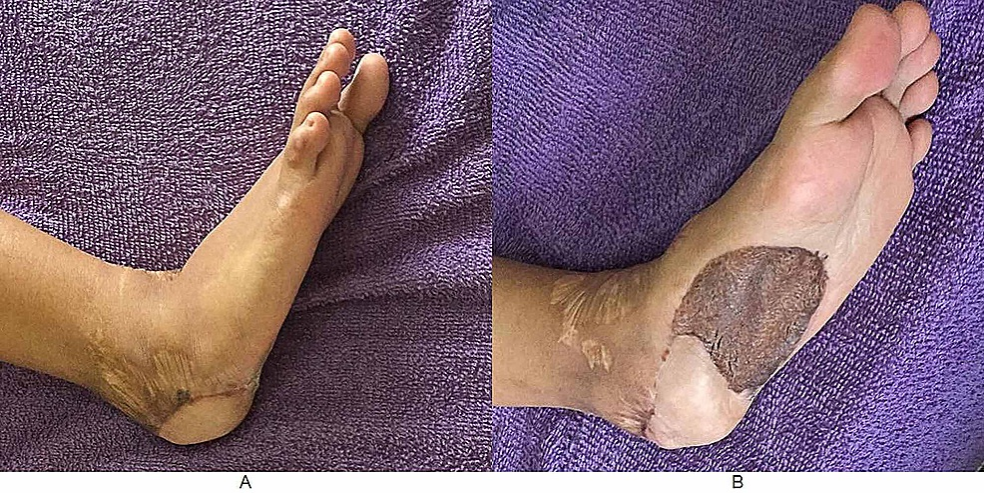
One year post operation

Postoperatively, the wound healed well. The flap was viable and supple (Figure 4, Figure 5). She was allowed gradual dorsiflexion two weeks after surgery. Maximum dorsiflexion was allowed three weeks after surgery. Full weight-bearing was allowed one month after surgery with splinting at night. One year postoperatively, she was able to ambulate and to weight-bear without any pain. Functional outcome was satisfactory.

## Discussion

The reconstruction of the heel remains a challenge due to the limited local tissue availability and also due to the special structural and functional characteristics of this particular region. Injury to the heel with complex tissue loss requires reconstruction aimed at restoring the form and function. The goal of resurfacing the heel pad is to replace like with like, restoring contour and function with durable, sensible, and stable vascularized tissue, along with providing minimal donor-site morbidity. The ideal plantar heel requires thick, durable, and glabrous skin to help resist shearing forces during walking or weight-bearing [3].

Owing to the unique structure of the heel pad, the heel pad plays an important role in shock absorption to the underlying bone during weight-bearing task. Loss of elasticity and changes in the thickness of the heel pad have been suggested as the main causes of heel pain [4]. Trauma to the heel can cause breakdown of fibrous tissue within the fat pad or degeneration of the heel fat pad, both of which can diminish its compressibility. A study performed by Iglesias et al. showed a relationship between fat pad thickness and heel pain, in which people with unilateral heel pain showed a significantly decreased thickness of the fat pad, regardless of gender [5]. In the presented case, even though there was no wound defect, there was a significant loss of heel pad over the affected leg. This resulted in heel pain during weight-bearing and was also esthetically unpleasant. For this reason, heel pad reconstruction was performed as a secondary procedure for better cosmetic outcome and functional benefits.

There are several techniques of reconstruction available to overcome the problem of heel coverage, including skin grafts, local advancement flaps, cross-leg flaps, island pedicle flaps, and microsurgical free flaps. In general, it can be noted that skin grafts can provide simple, yet effective coverage; however, it does not meet the requirements of this region. This may be the first choice in situations where there are small (<3 cm diameter) defects with well-vascularized granular base. In a cohort study by El-Shazly et al., split skin grafting option was applied for temporary coverage in two children before scheduling them for free flap later. Skin grafting was also used for permanent coverage in elderly individuals. Flaps, on the other hand, provide more durable and robust coverage in comparison to grafts, with lower rates of breakdown and recurrent ulceration, and they also provide a better cosmetic result in most cases [6].

The medial plantar (instep) area is a non-bearing area providing durable and stable skin with similar skin quality as the rest of the sole. Therefore, the instep medial plantar flap, harvested as a fasciocutaneous flap, is recognized as one of the preferred flaps, giving its advantage of local regional tissues. The flap offers thin, pliable glabrous tissue that is easily contoured and neurotized, providing potential for sensitization [7].

The medial plantar artery island flap was first described by Harrison and Morgan in 1981 to resurface plantar defects [8]. The advantages of this flap include providing skin coverage, which appears similar to the surrounding tissue, being a regional flap, therefore allowing reconstruction of like with like, providing globular skin with a fatty cushion and a fibrous septa fixed to the skin that are resistant to shear trauma and weight-bearing. Other than that, as this flap is innervated by the cutaneous branch of the medial plantar nerve, sensitization is possible, which is an important requirement for patient's ambulation [9]. Another advantage of medial plantar flap is the low donor-site morbidity [1]. As seen in our case, there were no long- or short-term donor-site complications in patient’s view. The donor site was covered with split-skin grafting, which healed well. The donor-site outcome appears esthetically less pleasing; nevertheless, it is an area that is easily concealed.

One disadvantage of the medial plantar flap is its limitation in size and coverage of deep and extensive cavity defects, especially in the case of trauma affecting the ipsilateral foot. Therefore, other options of reconstruction must be considered, such as muscle flaps or larger fasciocutaneous flaps [9].

## Conclusions

The medial plantar artery pedicle flap is a versatile and an effective method for reconstruction of weight-bearing heel soft tissue defects. It provides satisfactory esthetic, sensory, and functional recovery with minimal donor-site morbidity.
